# Molecular Detection and Phylogenetic Analysis of Selected Viral Pathogens in Wild Boar Populations of Russia

**DOI:** 10.3390/v18030307

**Published:** 2026-02-28

**Authors:** Valentina Rykova, Alina Komina, Irina Makhova, Elena Zhukova, Alexey Igolkin, Ivan Lavrentiev, Anton Yuzhakov

**Affiliations:** 1 Federal State Budget Scientific Institution “Federal Scientific Center VIEV” (FSC VIEV), 109472 Moscow, Russia; 2 Federal Centre for Animal Health, 600901 Vladimir, Russia

**Keywords:** wild boar, swine disease, porcine circovirus, porcine parvovirus, genetic diversity

## Abstract

The distribution and genetic diversity of economically significant pathogens, including porcine reproductive and respiratory syndrome virus (PRRSV), porcine circovirus type 2 (PCV2), porcine circovirus type 3 (PCV3), and porcine parvovirus 1 (PPV1), across extensive Russian territory within wild boars that serve as reservoirs remain poorly characterized. This study aimed to conduct a molecular epidemiological survey of these viruses in wild boar populations. The samples of 476 wild boars, collected across Russia between 2021 and 2025, were tested by PCR for the detection of viral genomes. While PRRSV was not detected, we found high detection rates for PCV2 (34.9%), PCV3 (35.5%), and PPV1 (25.4%). For PCV2, the co-circulation of two genotypes, PCV2b (5/53) and PCV2d (48/53), was observed. All 29 PCV3 sequences belonged to the PCV3a genotype. For PPV1, the presence of the PPV1a, PPV1b, and PPV1d genotype, as well as unclassified isolates, was shown. Co-infection of different viruses was detected: PCV2/PCV3 (16.0%), PCV2/PPV1 (6.9%), PCV2/PCV3/PPV1 (6.9%), and PCV3/PPV1 (4.4%). This is the first comprehensive study that demonstrates the wide dissemination and genetic diversity of PCV2, PCV3, and PPV1 within the wild boar population in Russia and highlights their role as a potential reservoir in viral evolution and spread.

## 1. Introduction

Industrial pig farming is an important part of the Russian Federation’s agricultural economy. However, outbreaks of infectious pig diseases can cause economic damage, including direct losses from animal mortality and reduced productivity, as well as indirect costs associated with vaccination, treatment, and trade restrictions. Among the viruses that cause such diseases, the porcine reproductive and respiratory syndrome virus (PRRSV), porcine circoviruses type 2 and 3 (PCV2 and PCV3, respectively), and porcine parvovirus 1 (PPV1) are of veterinary importance.

A significant factor that can complicate disease control is the wild boar population. As natural reservoirs, wild boars can influence the epidemiological situation, facilitating the evolution and spread of pathogens that cause swine infections. The interaction between wild boars and domestic pigs creates a persistent risk of infection transmission to domestic pigs [1]. The European wild boar (*Sus scrofa*) is an autochthonous species in Russia that inhabits the entire European part of the country, as well as the southern border up to the Far East. The wild boar population density in Russia is substantially lower than in European and American countries [1]. It is known for certain that the number of these animals has greatly decreased since the introduction and spread of African swine fever (ASF) in Russia, as well as due to culling as part of the fight against ASF outbreaks. The total number of wild boars in Russia was estimated at 209,000 animals in 2021 [2].

Investigation into the genetic diversity of viruses circulating in wild fauna is a critical component of veterinary surveillance, particularly in the context of potential cross-border transmission. Data on the prevalence of PRRSV in wild boar populations is limited. Genomes of PRRSV or specific antibodies are lacking or detected with low frequencies in studies from different countries [3,4,5]. A high detection rate has been described only in a study from Lithuania [6]. PPV1, PCV2, and PCV3 are widespread; their genomes or specific antibodies were detected in wild boar herds from many countries [7,8,9,10,11,12,13,14,15,16,17].

According to previous studies, PCV2, PCV3, and PPV1 are widespread in the wild boar population in several regions of the European part of Russia [18,19]. There is a lack of information about the prevalence and genetic diversity of these viruses in the North Caucasus, Urals, Siberia, and the Russian Far East. These territories occupy a significant part of Russia, border different countries, and are also associated with pork production. Consequently, the aim of our study was the detection and genotyping of PRRSV, PCV2, PCV3 and PPV1 in the wild boar population from different parts of Russia.

## 2. Materials and Methods

### 2.1. Sampling

**Figure 1:**
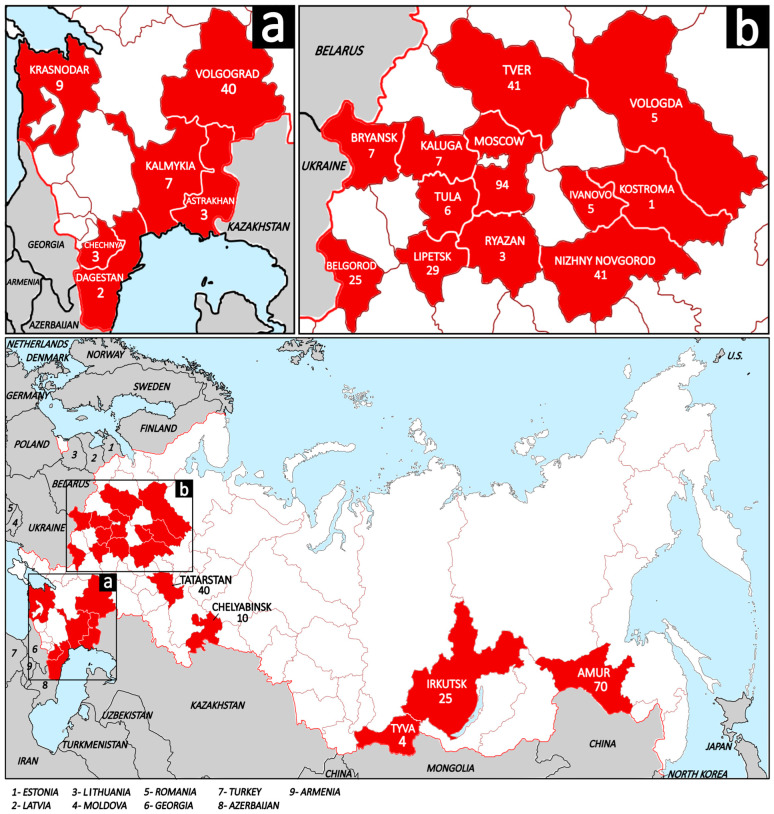
The map of the Russian Federation demonstrates the studied regions (shaded in red) and sampled wild boars per region. The white shading and brown lines represent the regions of Russia; the wide red line represents the state borders of Russia; the gray shading and black lines represent neighboring and nearby countries, and the blue shading represents water areas. (**a**) Regions of South European Russia and North Caucasus; (**b**) Regions of Central European Russia and North European Russia.

From 2021 to 2025 tissues samples (spleen, lymph node, and lungs) from 476 free-living European wild boars were collected. Detailed information about the samples is provided in the Supplementary Materials (Table S1). There was no provided information concerning the manifestation of clinical signs. All animals were hunted according to the hunting rules of Russia and sent for obligatory testing for ASFV (African swine fever virus) and CSFV (classical swine fever virus) in the government’s veterinarian laboratories at ARRIAH (Federal Centre for Animal Health, ARRIAH, 600901 Vladimir, Russia). The samples from wild boars were collected across 23 regions of Russia, spanning a vast territory including Central European Russia (Moscow, Tver, Kaluga, Tula, Ryazan, Bryansk, Lipetsk, Belgorod, Nizhny Novgorod, Ivanovo, Kostroma, and Tatarstan), North European Russia (Vologda), South European Russia (Krasnodar, Volgograd, Astrakhan, and Kalmykia), the North Caucasus (Chechnya and Dagestan), the Ural (Chelyabinsk), Siberia (Irkutsk and Tyva), and the Russian Far East (Amur) (Figure 1).

### 2.2. Nucleic Acids Extraction and PCR Assay

A piece of tissue (0.5–1 g.) was homogenized in a 5 mL sterile saline solution, aliquoted in 1.5 mL Eppendorf tubes, and stored at − 70 °C. Nucleic acids were extracted from the supernatant of tissue suspension using the commercial kit “RIBO-prep” (FBIS Central Research Institute of Epidemiology of Rospotrebnadzor, Moscow, Russia) following the manufacturer’s instructions and stored at −70 °C until further analysis.

All samples were previously tested for ASFV and CSFV via real-time PCR according to the recommendations of the WOAH and recognized as negative by the government’s veterinarian laboratories [20]. PCR assays for PRRSV, PCV2, and PPV1 detection were performed using commercial qPCR kits (Vetbiochem, Moscow, Russia) following the manufacturer’s instructions. Detailed information about the primers used in the current study is provided in the Supplementary Materials (Table S3). For PCV3, the PCR assay developed by Palinski et al. was performed [21]. The PCR products were analyzed by electrophoresis in 1% agarose gel prepared in a tris–acetate buffer solution (pH 8.0) with the addition of ethidium bromide (0.5 µg/mL) and the following gel examination under ultraviolet light.

This study summarizes the results of previous and current investigations. As part of our study, 108 of 476 wild boars from the Moscow, Tver and Belgorod regions were tested for the presence of different viruses and published previously [18,19].

### 2.3. Sequencing and Bioinformatic Analysis

Samples with the lowest Ct-values in qPCR in the case of PCV2 and PPV1, or the best quality during electrophoresis in the case of PCV3, were subjected to sequencing and phylogenetic analysis. Sequencing was performed using previously designed primers [19,22,23] (Table S3). The resulting overlapping fragments covered ORF2 (coding capsid protein VP2) of PPV1, the complete genome or ORF2 (coding capsid protein) of PCV2 and the complete genome of PCV3. PCR products were purified from gel using the Cleanup Standard Kit (Evrogen, Moscow, Russia). Sanger sequencing was performed in both directions with the previously mentioned PCR primers using the Big Dye 3.1 Terminator Cycle Sequencing Kit (Applied Biosystems, Foster City, CA, USA) on the ABI PRISM 3130 Genetic Analyzer (Thermo Fisher Scientific, Carlsbad, CA, USA). The obtained sequence chromatograms were analyzed, trimmed and assembled into consensus sequences using SeqMan Lasergene 11.1.0 software (DNASTAR, Madison, WI, USA).

The phylogenetic analysis was performed using MEGA 7.0 software [24]. The obtained sequences were aligned by the MUSCLE algorithm. Phylogenetic dendrograms were plotted using the maximum likelihood (ML) method and the GTR (G + I) model. The topology evaluation was performed by 1000 bootstrap replications. Pairwise distances were calculated using the Distance Estimation analysis method Maximum Composite Likelihood by MEGA 7.0 software.

A comparative analysis was conducted to evaluate the main immunodominant epitopes on the capsid protein of PPV1 and PCV2. The nucleotide sequences of ORF2 were translated into amino acids and aligned using the ClustalW algorithm in BioEdit 7.0 software.

### 2.4. Statistical Analysis

Statistical analysis was conducted using Past 4.17 software. Differences in the detection rates of various combinations of the infections PCV2, PCV3, and PPV1 were investigated using the exact Fisher criterion by pairwise comparisons. Results with a *p*-value of <0.05 were considered statistically significant.

## 3. Results

### 3.1. Distribution of Viruses

During 2021–2025, we collected samples from 476 wild boars from 23 regions of Russia. All samples tested negative for PRRSV. The PCV2, PCV3, and PPV1 genomes were detected in samples from different regions (Table S2).

The PCV2 genome was detected in wild boars across 18/23 regions, excluding Dagestan, Chechnya, Kalmykia, Kostroma, and Tyva, and the overall detection rate was 34.9% (166/476). The lowest PCV2 detection rate, at 5.7%, was observed in Amur, while the highest, at 76.0%, was in Belgorod. Genomes of PCV3 were detected in 17/23 regions, excluding Dagestan, Chechnya, Kostroma, Tatarstan, Irkutsk, and Tyva. The detection rate across regions for PCV3 was 35.5% (169/476). The lowest detection frequency, at 10.0%, was noticed in wild boar populations within Volgograd and Chelyabinsk, whereas the highest frequency, reaching 100.0%, was recorded in Ivanovo. Genomes of PPV1 were detected in 16/23 regions, excluding Dagestan, Chechnya, Ryazan, Kaluga, Kostroma, Irkutsk, and Tyva. The average detection rate among regions was 25.4% (121/476). Vologda showed the highest detection rate (60.0%), followed by Krasnodar (44.4%) and Bryansk (42.9%). The lowest values were observed in Tatarstan—5.0%—and Amur—1.4%. Over a five-year period from 2021 to 2025, PCV2, PCV3, and PPV1 were identified in samples originating from the Moscow region, though with varying frequencies.

**Figure 2:**
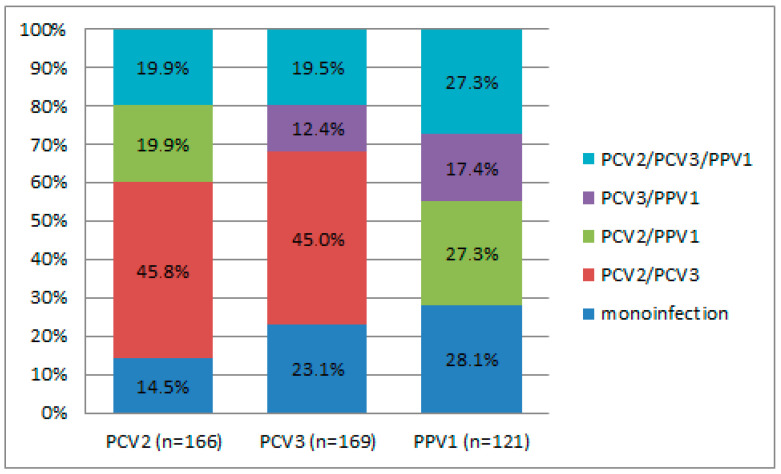
Distribution of positive cases of mono-, dual, and triple infections. The “n” means the total number of wild boars in which a particular virus was detected.

Different combinations of virus co-infections were observed in 34.2% (163/476) of wild boars; the highest rate was noticed for PCV2/PCV3 (16.0%) (Table S2). The PCV2/PPV1 dual infection and the triple infection of PCV2/PCV3/PPV1 were each identified in 6.9% of the wild boar population. In contrast, the PCV3/PPV1 combination was less common, occurring in just 4.4% of wild boars. When considering the distribution of positive cases for mono-, dual-, and triple infections with PCV2, PCV3, and PPV1, statistically significant differences were noted for the PCV2/PCV3 dual infection (*p* < 0.001) in the case of both PCV2 (45.8%) and PCV3 (45.0%) (Figure 2). The detection rates of PPV1 in combination with other viruses were not statistically significant. Upon assessing geographic patterns in the frequency of co-infections, no regions with a significant predominance of either combination were identified. However, a high frequency of PCV2/PCV3 detection in the Tver region (*p* = 0.068) is noteworthy.

### 3.2. PCV2 and PCV3 Sequence Analysis

**Figure 3:**
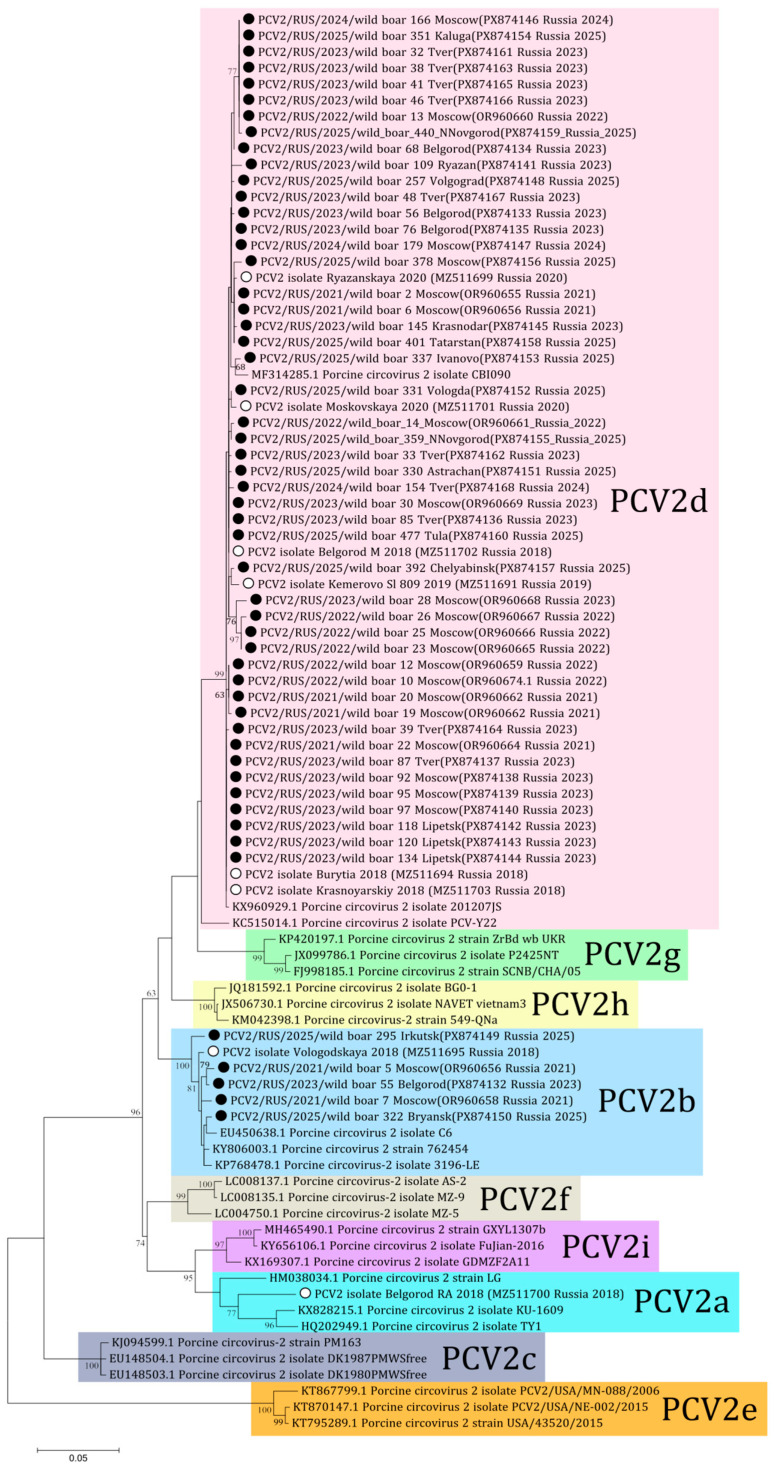
Phylogenetic tree of ORF2 of PCV2. The obtained sequences are designated by black circles ⚫. The sequences previously obtained from Russian domestic pigs are designated by white circles ⚪ [23]. Bootstrap is enabled for nodes where it is above 60%. The fill colors represent the different PCV2 genotypes listed to the right of each clade.

In this study eight complete genomes and 29 sequences of ORF2 for PCV2 were derived (PX874132-PX874168). Aside from these, 16 sequences of PCV2 obtained from wild boars in the Moscow region were published previously (OR960655-OR960669) [18]. In total, complete genome sequences of PCV2 were obtained from 23 samples and ORF2 sequences were received from 30 samples of wild boars from 17 regions. Following phylogenetic analysis, the majority of the sequences (48 out of 53) were determined to be of the PCV2d genotype, with the remaining five sequences being classified as PCV2b (Figure 3). The PCV2b genotype included sequences received from Moscow in 2021 (wild boars 5 and 7), Belgorod in 2023 (wild boar 55), and Irkutsk and Bryansk in 2025 (wild boars 295 and 322 respectively). Comparative analysis showed 93.1–100% nucleotide similarity for ORF2 of PCV2 among the sequences from Russian wild boars.

**Figure 4:**
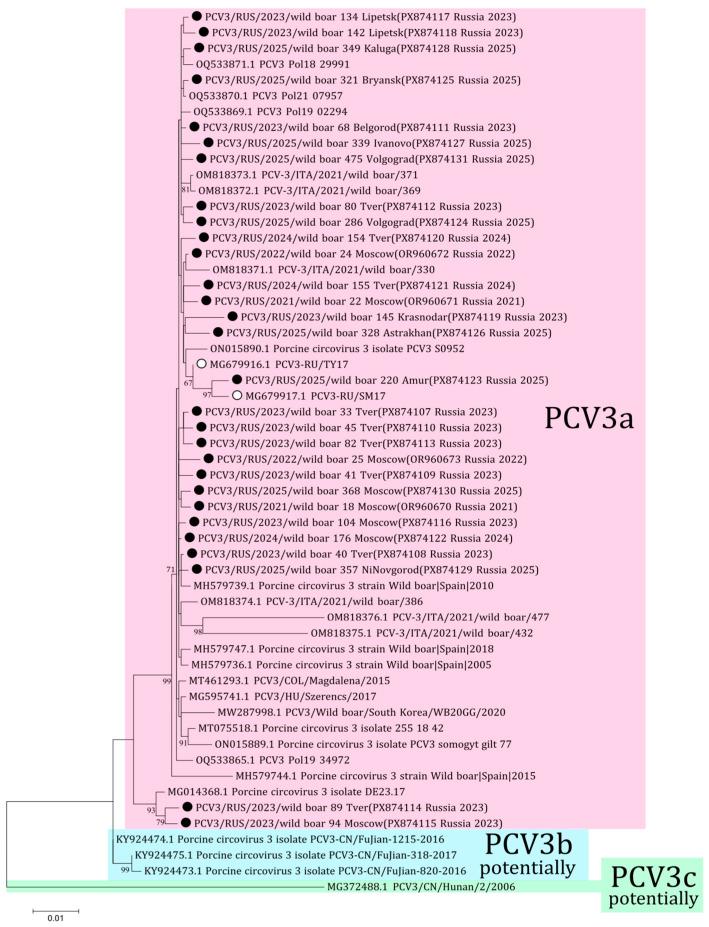
Phylogenetic tree of PCV3. The obtained sequences are designated by black circles ⚫. The sequences previously obtained from Russian domestic pigs are designated by white circles ⚪ [22]. Bootstrap is enabled for nodes where it is above 60%. Genotyping of PCV3 is given in accordance with the genotyping criteria proposed by Franzo et al. [25]. The fill colors represent the different PCV3 genotypes listed to the right of each clade.

The phylogenetic analysis of PCV3 received from Russian wild boars was performed using 25 complete genome sequences (PX874107-PX874131) from 12 regions obtained in this study and four complete sequences (OR960670-OR960673) from the previous study [18]. All 29 sequences belonged to the PCV3a genotype (Figure 4). The sequences from Tver and Moscow (PX874114 and PX874115, respectively) formed the distant clade with the German isolate (MG014368) from 2015. Comparative analysis showed 97.5–99.8% nucleotide similarity among the sequences from Russian wild boars.

The comparative analysis of the capsid protein sequences revealed amino acid substitutions between the PCV2a vaccine strain (AF264042) and the studied isolates across all four known antibody recognition domains. Furthermore, amino acid differences were identified within all previously characterized decoy epitopes identified by Trible et al. and Ilha et al. (Figure S1) [26,27].

### 3.3. PPV1 Sequence Analysis

During our study, 21 partial sequences of PPV1 were obtained (PX843310-PX843330). In addition, 17 (PQ757578-PQ757594) sequences were published previously [19]. In total, sequences were received from 16/23 regions of Russia. Comparative analysis showed 98.3–100% similarity among all 38 nucleotide sequences.

**Figure 5:**
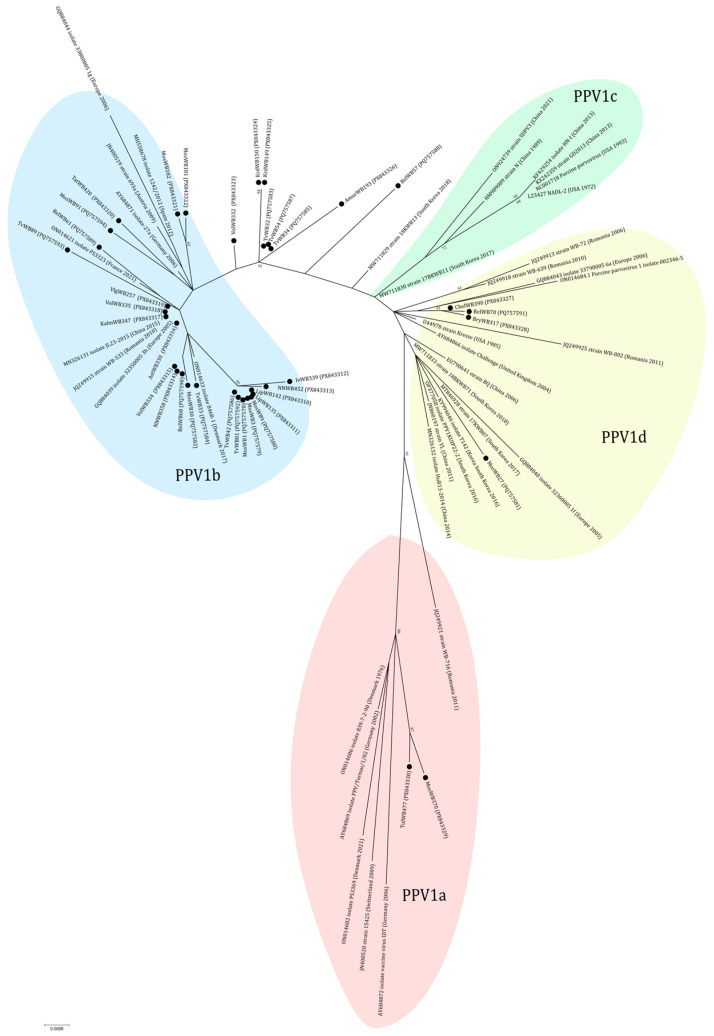
Phylogenetic tree of PPV1. The obtained sequences are designated by black circles ⚫. The fill colors represent the different PPV1 genotypes listed in the fill area for each clade. The clusterization of PPV1 strains is given in accordance with the classification of Vereecke et al. [28].

According to the results of phylogenetic analysis, the majority (24 out of 38) of sequences are classified within the PPV1b cluster (27a-like group) (Figure 5). In this cluster some sequences from Central Russia (MosWB1, MosWB3, MosWB9, TvWB42, TvWB81, LipWB135, LipWB142, IvWB339, and NNWB452) formed a separate branch with high bootstrap support. The cluster PPV1d (Kresse-like group) included four sequences from Central Russia and the Urals: MosWB27, BelWB70, BryWB317, and ChelWB390. Three of them (BelWB70, BryWB317, and ChelWB390) formed a separate branch (nucleotide identity of 99.78–100%). The cluster PPV1a (IDT-like group) included two sequences from wild boars from the bordering Moscow (MosWB370) and Tula (TulWB477) regions. The isolates TvWB32, TvWB34, TvWB54, BelWB57, KrdWB149, KrdWB150, AmurWB193, and VolWB332 received from European Russia and the Russian Far East cannot be assigned to any of the clusters. They formed a separate branch and had amino acid substitutions Ala414 → Ser and Ser436 → Thr typical for 27a-like strains and not including Gln228 → Glu and Glu419 → Gln as well as Kresse-like strains and the IDT vaccine strain.

Among VP2 epitopes previously discovered by Sun et al., amino acid substitutions were found in (228) QQITDA (233) in positions 233 (S → T) and 228 (Q → E) [29].

## 4. Discussion

Studying the circulation of porcine viruses in wild boar populations, including analysis of their prevalence and genetic diversity, is a critical component of epidemiological surveillance implemented in many countries to reduce risks to livestock biosecurity and wildlife health. The current study includes both the European and Asian parts of the Eurasian continent. Some of the studied regions have a land border with different countries: Bryansk—Ukraine and Belarus; Belgorod—Ukraine; Krasnodar and Chechen—Georgia; Dagestan—Georgia and Azerbaijan; Astrakhan, Volgograd and Chelyabinsk—Kazakhstan; Tyva—Mongolia; and Amur—China. The extensive geographical scope of this study provides valuable data for our national context and also offers a unique opportunity to investigate the potential for cross-border viral transmission. By “cross-border” transmission, we mean the free movement of wild boars across state borders.

Data on the prevalence of PRRSV in wild boar populations is limited. Only 1.7–2.5% of feral swine sera contained PRRSV antibodies in different studies from the USA [4,15]. In France, the seroprevalence in free-living and farmed boars was estimated to be 1.3% and 8.3%, respectively [3]. However, in Lithuania, which shares a border with Russia, the genetic material of PRRS viruses was found in an average of 19% of the samples obtained from wild boars [6]. In Germany, where wild boar herds have a high density, 15.9% of animals were positive for PRRSV. Furthermore, both species of the virus were present in the infected wild boars [30]. In numerous studies, neither PRRSV genomes or specific antibodies have been detected, indicating that PRRSV-positive samples from wild boars are a rarity rather than a common occurrence [5,8,31,32]. Our results regarding the lack of PRRSV genomes is consistent with this global trend and aligns with prior research conducted in Russia [33]. Despite the fact that the PRRS virus is widespread among domestic pigs and transmitted by airborne pathogens over long distances, it is not common among wild boars [34]. This may indicate not only a limited contact between wild boars and domestic pigs, but also that wild boars are less susceptible to PRRSV than pigs. In any case, domestic pigs are more likely to be the reservoir of the PRRSV, and wild boars have almost no role in the spread and evolution of this virus [1].

In the current study, genomes of PCV2 and PCV3 were detected in 34.9% (166/476) and 35.5% (169/476) of wild boar samples, respectively. Studies from other countries have also reported the presence of PCV genomes or antibodies in wide detection ranges in wild pig populations worldwide. For example, PCV2 was identified in Poland at 75.6%, and PCV3 at 37.8%; in Ukraine, PCV2 occurred in 31.8%; in the USA, prevalence ranged from 25.3% to 71.7% across various studies; in Japan, PCV2 was found in 8.3% and PCV3 in 16.8%; and in China, PCV2 appeared in 58.3% and PCV3 in 10.9% of cases [12,13,14,15,16,17,35]. The distinctions in the detection rates could stem from differing methodologies, sample types, and wild boar population densities across counties.

The co-circulation of different PCV2 genotypes is typical and reported in many countries [12,14,36]. In Russia, PCV2d (48/53) predominates among wild boars, whereas PCV2b (5/53) was detected at a low frequency in geographically separate regions throughout different study years. Among the regions where PCV2b was detected, Belgorod and Bryansk border other countries. Both of these regions border Ukraine, and Bryansk also borders Belarus. There is no data on the genotypic diversity of PCV2 in the wild boar population in Belarus. Genotypes b, a, and f were detected in wild boar samples from 2012 in Ukraine, PCV2b being the predominant genotype [14]. Despite the fact that there are no recent data on bordering countries, the possibility of cross-border transmission cannot be ruled out, as wild boars can freely move across national borders. There is no current data about the actual PCV2 genotypes circulating in Russian domestic pigs. In the previous work, PCV2a (1/14), PCV2b (1/14) and PCV2d (12/14) genotypes were detected in samples from pigs in 2018–2020 [23]. Some regions of the current study overlap with previous pig herd studies: Vologda (pigs 2018 PCV2b, wild boars 2025 PCV2d), Belgorod (pigs 2018 PCV2a and PCV2d, wild boars 2023 PCV2b, PCV2d), Moscow (pigs 2020 PCV2d, wild boars PCV2b and PCV2d in 2021, then 2022–2025 PCV2d), Ryazan (pigs 2020 and wild boars 2023 PCV2d). Vaccination against PCV2 based on the PCV2a genotype vaccine is a standard preventive measure in Russian pig farms. The absence of PCV2a in wild boars, which are not exposed to vaccine pressure, may suggest a competitive advantage or higher virulence of the PCV2d and PCV2b genotypes in non-vaccinated populations. However, there is no data about frequent interaction or viral exchange between wild boars and pig farms in Russia. Significant variation in immunodominant epitopes compared to the vaccine strain in non-vaccinated wild boars indicates ongoing viral evolution and highlights the need to study the direction of transmission. There are not significant differences between the wild boar sequences from the current study and domestic pig sequences from previous studies in Russia into neutralizing epitopes. Consequently, these data highlight the necessity for further investigation of PCV2 epidemiology in vaccinated pig herds.

The genotyping diversity of PCV3 is a controversial issue. According to the previously proposed criteria for genotyping, only PCV3a is currently distinguished [25]. Genotype PCV3a was detected in samples from domestic pig herds in two regions of Russia in 2017 [22]. All sequences of the complete PCV3 genome from our study belong to the PCV3a genotype with 97.5–99.8% nucleotide identity. Two sequences from Tver (wild boar 89, PX874114) and Moscow (wild boar 94, PX874115) showed a high nucleotide difference with other isolates from Russian wild boars, up to 2.5% by complete genomes. These sequences form the separate branch with the isolate from Germany (MG014368) with high bootstrap support. However, the percentage of nucleotide difference and the small number of samples do not allow us to identify a new genotypic group according to the genotyping criteria proposed for PCV3. The presence of these different sequences may be associated with the formation of a new genetic group, as well as with the presence of low-fitness strains in the wild boar population in Russia. The accuracy of the latter assumption suggests that similar isolates from Germany were gathered in 2015, nearly a decade prior to our investigation. Furthermore, both of these isolates from Russia were sampled in 2023 in border regions and have not been detected since; thus, it is possible that wild boars 89 and 94 were animals from one herd.

In our study, the detection rate of the PPV1 genome was 25.4%. Among the countries that are geographically close to Russia, the genomes or antibodies of PPV1 in wild boars were detected in Finland (46.5%), South Korea (5.4%), and Eastern Europe: Ukraine, Romania (5.2%), Serbia (56%), and Croatia (41.6%) [7,8,9,10,11,37]. In a previous study from Russia conducted between 2002 and 2005, PPV1 antibodies were found in 59.1% of wild boar samples collected from the Moscow, Vladimir, Tver, and Belgorod regions. In 2007 seroprevalence was 45% in wild boars from the Moscow region [7,38]. The lower PPV1 detection rate observed in this study is primarily attributed to variations in sample type and detection methods. According to the classification by Vereecke et al., the 38 sequences we obtained from 16 regions of the country were classified into four groups: PPV1a, PPV1b, PPV1d and unclassified isolates.

The majority of the sequences (24 out of 38) belong to the PPV1b cluster. These 27a-like isolates have been received from 11 studied regions from Southern (Astrakhan, Kalmykia, and Volgograd), Central (Moscow, Tver, Lipetsk, Ivanovo, Nizhny Novgorod, Belgorod, and Tatarstan), and Northern (Vologda) parts of European Russia. In the PPV1b cluster, nine sequences from Central Russia (Moscow, Tver, Lipetsk, Ivanovo, and Nizhny Novgorod) formed a monophyletic branch with high bootstrap support. The presence of similar viruses in this part of Russia may be due to their geographical proximity and the transmission of the virus between wild boar herds during seasonal migrations. PPV1d (Kresse-like group) was sequenced from samples of wild boars from the Moscow, Belgorod, Bryansk, and Chelyabinsk regions. The last area on this list is geographically remote from others and represents the Ural part of Russia. The PPV1a cluster includes sequences from the bordering Moscow (MosWB370) and Tula (TuWB477) regions. The co-existing PPV1a, PPV1b and PPV1d isolates were observed in the Moscow region. Particular attention must be given to the identification of the distinct group of isolates TvWB32, TvWB34, TvWB54, BelWB57, KrdWB149, KrdWB150, AmurWB193, and VolWB332, characterized by amino acid substitutions typical of Kresse-like (Gln228 and Glu419) and 27a-like (Ser414 and Thr436) strains. The sequences were received from Central (Tver, Belgorod), Southern (Krasnodar), and Northern (Vologda) European Russia and the Far East (Amur) of Asian Russia. This suggests the possible emergence of a new or transitional subline. In addition, the detection of such unclassified isolates is interesting, since it was previously reported that a similar strain (the capsid profile contained characteristic mutations for the PPV1b and PPV1d clades) demonstrated increased antibody titers for the tested antisera [28]. Such “mixed” isolates can be considered for vaccine development and require further in vitro studies. In Russia, vaccination against PPV1 is a standard preventive measure. Until recently, the market was dominated by foreign vaccines such as FarrowSure Gold B (Zoetis Inc., Parsippany-Troy Hills, NJ, USA) and Eryseng Parvo/Lepto (Hipra, Amer, Catalonia, Spain), based on ‘classical’ strains like NADL-2, IDT etc. Currently, the vaccination strategy relies more heavily on Russian vaccines, but the data about the genomic sequences or exact origin of these strains is not available. Generally, the concurrent circulation of these various genotypes may pose challenges for controlling PPV1, and underscores the importance of surveillance to monitoring how PPV1 evolves and spreads among wild boar populations, which could serve as viral reservoirs. We did not find PPV1c (NADL-2-like) isolates in samples from Russian wild boars, despite the fact that it is known to exist in neighboring China. This may be due to the small number of sequences from Asian Russia. Previously the genetic diversity of PPV1 in the wild boar population was described in Romania. It is assumed that PPV1 is more diverse in wild boars than in pig herds, resulting from lower population density and a lack of vaccine pressure [9]. A further study about actual virus strains circulating in Russian pig farms is necessary to improve knowledge about PPV1 diversity in our country.

Amino acid substitutions were identified within one of the three neutralizing antibody epitopes reported by Sun et al. in VP2 [29]. The epitope (228) QQITDA (233), located on the viral particle’s surface, is not conserved; a common single mutation at residue 233 (S → T) was observed in an isolate from the Tula (TulWB477) region. In addition, some sequences had 228 (Q → E) substitution typical for 27a-like strains. Identifying single substitutions within solely one epitope may indicate that PPV1 variability among wild boars has no impact on these antigenic sites.

The co-infection cases with PCV2, PCV3 and PPV1 were detected in both domestic pig and wild boar populations. Thus, the 22.3% of PCV2/PCV3 co-infection was detected in wild boar herds in the Campania region of Italy [39]. Furthermore, in wild boars from the Sardinia region of Italy, PCV2/PCV3 dual infection was not observed at all, PCV3/PPV1 was detected in only one animal (4.17%), while triple infection (PCV2/PCV3/PPV1) was found in the majority of the cases (95.8%) [40]. Some studies suggest a synergistic effect of these viruses and report cases of PCV2/PPV1 and PCV2/PCV3 [41]. In the current study, low percentages of the co-infections PCV2/PCV3 (16.0%), PCV2/PPV1 (6.9%), PCV3/PPV1 (4.4%), and PCV2/PCV3/PPV1 (6.9%) were detected. In addition, as no significant differences were found between regions. The statistically significant differences were noted for the PCV2/PCV3 dual infection (*p* < 0.001) which may confirm the synergistic effect of these viruses.

There were some limitations to our study: a small number of samples from some regions (Kostroma—1, Dagestan—2, Ryazan—3, Chechnya—3, Astrakhan—3); a lack of samples from some regions which border other countries; a different sample type; and the impossibility of managing samples’ storage conditions prior to laboratory admission. Thus, the observed genotype distribution, lack of positive cases in certain areas, and broad variation in virus detection frequencies may stem from both regional factors and study limitations.

The presence of genetic diversity for pathogens of veterinary significance, such as PCV2 and PPV1, might suggest complex interactions between viral strains found in wild boars and those in domestic pigs. The vaccination pressure on domestic pigs might drive viral adaptation and lead to the emergence of novel genetic lineages. On the other hand, the small DNA genomes of *Circoviruses* and *Parvoviruses* exhibit a rapid evolutionary rate and capacity for recombination, potentially leading to the spontaneous emergence of novel genetic variants [28,42]. The genetic variation observed in wild boars might stem from several factors: the introduction of virus strains appeared as a consequence of vaccination pressure in pigs, the persistence of earlier viral types’ absence in vaccinated animals and the emergence of novel distinct mutated variants. Given the aforementioned, all the factors are involved and indicate that wild boars may serve as a reservoir, capable of hosting extensive genetic diversity among the studied viruses. The direction of viral transmission is a debatable topic and a focus of our ongoing research [1]. We hypothesize that due to the high standards of biosecurity in Russian pig farms, transmission from wild boars to pigs is less likely than transmission from pigs to wild boars through pig products. Consequently, in order to better understand the key factors behind this phenomenon, it is crucial to investigate the prevalence and genetic diversity of these viruses in Russian domestic pig herds. Analyzing this information would enable comparative analysis and assist in developing stronger, more robust hypotheses.

## Data Availability

The data presented in the study are deposited in the NCBI GenBank repository, accession numbers OR960655-OR960674, PQ757578-PQ757605, PX843310-PX843330 and PX874107-PX874168.

## Supplementary Materials

The following supporting information can be downloaded at: https://www.mdpi.com/article/10.3390/v18030307/s1, Figure S1: Immunodominant regions of the PCV2 capsid protein; Table S1: Wild boar sample information; Table S2: Distribution of PCV2, PCV3 and PPV1 in different regions of Russia in 2021–2025; Table S3: PCR primers and commercial test-system using in current study for detecting and sequencing of viruses.

## Abbreviations

The following abbreviations are used in this manuscript:

ARRIAH	Federal Centre for Animal Health
ASFV	African swine fever virus
CSFV	Classical swine fever virus
PCV2	Porcine circovirus type 2
PCV3	Porcine circovirus type 3
PPV1	Porcine parvovirus 1
PRRSV	Porcine reproductive and respiratory syndrome virus
WOAH	World Organisation for Animal Health
