# Supplementary material for: Molecular Detection and Phylogenetic Analysis of Selected Viral Pathogens in Wild Boar Populations of Russia
Source: Viruses. 2026 Feb 28;18(3):307. doi: 10.3390/v18030307 (PMC13030787; doi:10.3390/v18030307)
Supplement: Supplementary file 1 [file viruses-18-00307-s001.zip › Supplementary/Figure S1.docx]

**
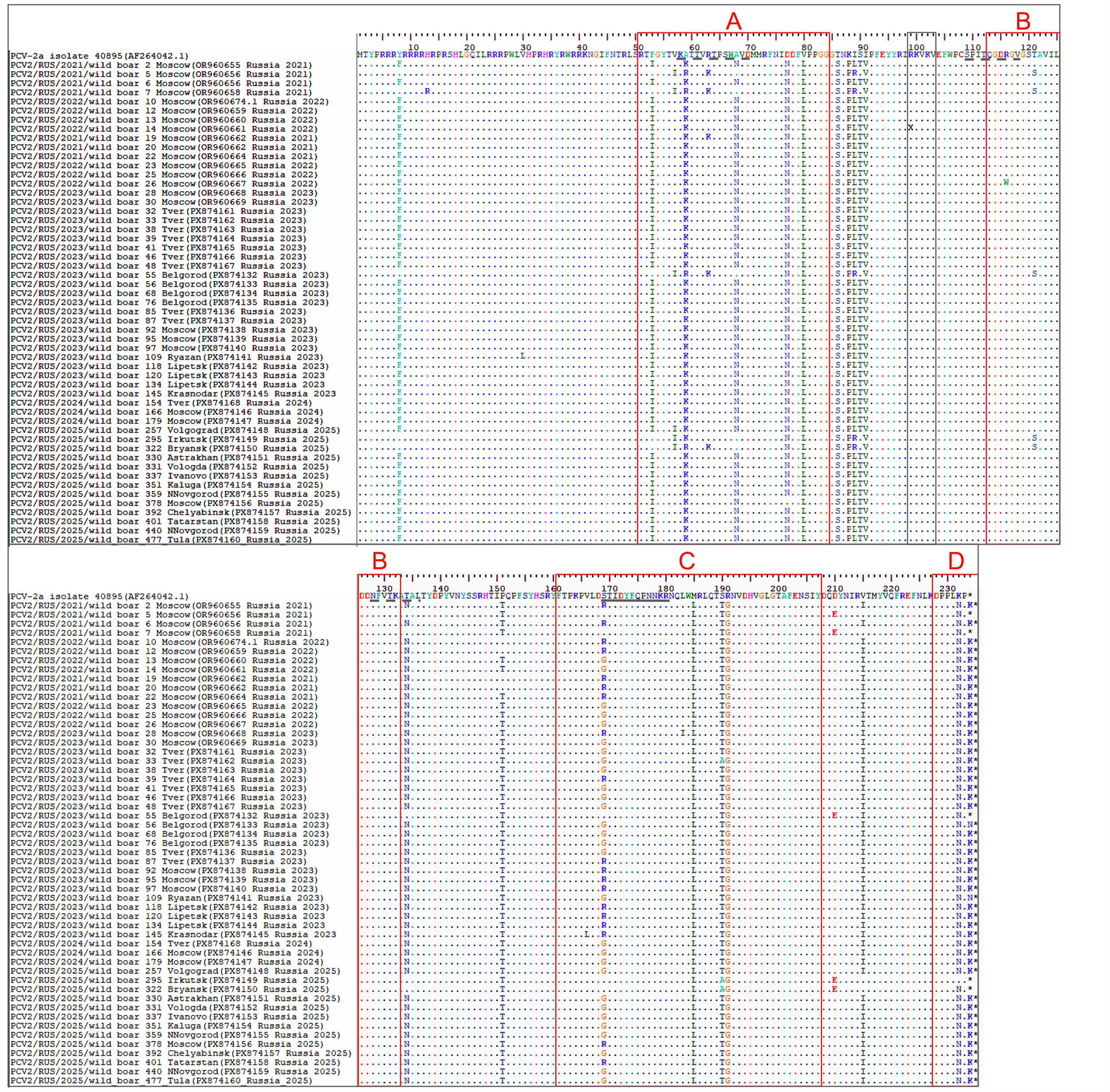
**

**Figure S1.** Immunodominant regions of the PCV2 capsid protein. Sequence alignment of the PCV2a vaccine strain (AF264042) and sequences from current study. Red boxes - four major immunodominant regions (A-D), grey box - putative heparin-binding motif, solid line – decoy epitope identified by Trible et al., dotted line – putative decoy epitopes identified by Ilha et al.
